# Tackling Antibiotic Resistance: Influence of Aliphatic Branches on Broad-Spectrum Antibacterial Polytriazoles against ESKAPE Group Pathogens

**DOI:** 10.3390/pharmaceutics14112518

**Published:** 2022-11-19

**Authors:** Cristian Rangel-Núñez, Inmaculada Molina-Pinilla, Cristina Ramírez-Trujillo, Adrián Suárez-Cruz, Samuel Bernal Martínez, Manuel Bueno-Martínez

**Affiliations:** 1 Departamento de Química Orgánica y Farmacéutica, Facultad de Farmacia, Universidad de Sevilla, C/Profesor García González 2, 41012 Sevilla, Spain; 2 Servicio de Microbiología, Hospital Universitario Virgen de Valme, 41014 Sevilla, Spain

**Keywords:** ESKAPE, cationic polymer, antibacterial polymer, antibiotic resistance, click polymerization

## Abstract

One of the most important threats to public health is the appearance of multidrug-resistant pathogenic bacteria, since they are the cause of a high number of deaths worldwide. Consequently, the preparation of new effective antibacterial agents that do not generate antimicrobial resistance is urgently required. We report on the synthesis of new linear cationic antibacterial polytriazoles that could be a potential source of new antibacterial compounds. These polymers were prepared by thermal- or copper-catalyzed click reactions of azide and alkyne functions. The antibacterial activity of these materials can be modulated by varying the size or nature of their side chains, as this alters the hydrophilic/hydrophobic balance. Antibacterial activity was tested against pathogens of the ESKAPE group. The P3TD polymer, which has butylated side chains, was found to have the highest bactericidal activity. The toxicity of selected polytriazoles was investigated using human red blood cells and a human gingival fibroblast cell line. The propensity of prepared polytriazoles to induce resistance in certain bacteria was studied. Some of them were found to not produce resistance in methicillin-resistant *Staphylococcus aureus* or *Pseudomonas aeruginosa*. The interaction of these polytriazoles with the *Escherichia coli* membrane produces both depolarization and disruption of the membrane.

## 1. Introduction

Since penicillin was discovered and systematically produced, infectious diseases were relegated to noncommunicable diseases, such as cardiovascular disease or cancer, both leading causes of death. The discovery of penicillin and subsequent new broad-spectrum antibacterial drugs marked the beginning of a new era. However, the ability of bacteria to develop mutations and the overuse of those antibiotics have induced the appearance of bacterial strains that could resist drugs to which they were originally sensitive [1].

In this context, there is a group of antibiotic resistant pathogens that exhibit high virulence, especially in nosocomial infections acquired by immunosuppressed patients, known as ESKAPE pathogens. This acronym represents six bacterial genres or species: *Enterococcus faecium*, *Staphylococcus aureus*, *Klebsiella pneumoniae*, *Acinetobacter baumanii*, *Pseudomonas aeruginosa*, and *Enterobacter* spp. These bacteria acquired resistance determinants to avoid antibiotic effects, such as the production of enzymes that can degrade bioactive compounds, the development of biofilms that create a mechanical and biochemical wall, or the overproduction of efflux pumps in the outer membrane that do not allow drugs to reach the cytosol at therapeutic concentrations [2]. These resistance genes are a significant risk to public health, and the World Health Organization (WHO) has recommended establishing research strategies focusing on the discovery and development of new antibiotics active against multidrug resistant bacteria [3,4,5]. Today, the emergence of multidrug and pandrug-resistant bacteria has become a challenging issue to address, as antibiotic-resistant bacteria are predicted to cause 10 million deaths by 2050 [6].

The challenge consists in developing well-tolerated, effective, and low-toxicity antimicrobial agents. In this regard, the design and synthesis of antimicrobial macromolecular systems, polymers, and copolymers have the potential to become a long-term solution to tackle these hazardous bacteria. This kind of structure is less likely to induce resistance than other antibiotics such as penicillin derivatives, cephalosporins, or carbapenems [7]. Antimicrobial polymers also have some important advantages over other structures like antimicrobial peptides because the polymers have greater stability, are cheaper, and are easy to prepare. They have also been proposed as next-generation biocides, as their preparation is more environmentally friendly [8].

Not only are antimicrobial polymers useful as antibiotics, but they also have other uses, such as surface coatings, preventing adhesion and colonization of bacteria on biomedical devices, and other relevant surfaces [9]. Briefly, antimicrobial polymers contain at least two types of domains: hydrophobic domains (aliphatic, aromatic, etc.) and positively charged domains (ammonium, thiazolium, guanidinium, etc.), also known as cationic domains, that can be found at side chains [10,11], at the backbone [12,13], or both [14]. On this basis, there are some modulating parameters that seem to play a key role in the efficacy of antibacterial polymers: amphipathicity, cationicity, molecular weight, chain length, and functional group [15]; however, the architecture of the polymer also seems to play an important role in the efficacy of those polymers. For example, recently, Liu et al. [16] prepared polymers containing subunits that differ in their stereochemistry, showing that these differences could lead to differences in the biological properties of the polymers. In addition, differences between regiospecific and non-regiospecific polymers obtained through azide-alkyne ‘click’ chemistry [12] or variations in topology and flexibility of polymers [13] influence activity against different microorganisms.

In recent decades, a wide variety of polymeric materials based on poly(1,2,3-triazole) have been synthesized using click chemistry. For the preparation of these materials, the cycloaddition reaction between azide and alkyne groups, catalyzed by copper (CuAAC) [17,18,19,20], which is highly efficient and regiospecific, or carried out in the absence of a metal catalyst, such as those promoted by tension (strain promoted alkyne-azide cycloaddition, SPAAC) [21,22,23,24], or by heat (Huisgen cycloaddition) in the presence [25,26] or absence of solvent [27,28,29], has been widely used. The growing interest in click chemistry is evidenced by the large number of applications that can be found in very different areas of research, for example, its use for the controlled synthesis of macromolecules of different structures [30,31,32,33,34,35], for the preparation of biobased materials [36,37] and hydrogels [38,39], in glycoscience [40], in biomedicine [41,42], or in industry [43].

The present work describes the synthesis, characterization, and in vitro antibacterial activity of new polycationic polymers with side chains of different sizes and, in some cases, of a different nature, with the purpose of modulating and improving their antibacterial properties and biocompatibility. These polymers have been obtained through azide-alkyne ‘click’ polymerization, by CuAAC and solvent- and metal-free 1,3-dipolar cycloaddition reactions, using, on the one hand, a derivative of tetraethylenepentamine as diyne monomer, which will give rise to three protonatable amines in the repeating unit of polymers after deprotection, and on the other hand, a diazido monomer that was synthesized by alkylation of the alcohol functions present in 1,12-diazido-4,9-dioxadodecan-2,11-diol using different alcohols and alkyl halides.

The synthesized polycationic polymers were evaluated against ESKAPE pathogens. In addition, their hemolytic activity, toxicity against human cell lines, membrane permeabilization/disruption, and induced resistance were tested.

## 2. Materials and Methods

### 2.1. Materials

The chemicals, including fluorescent probes, were all used as purchased from MilliporeSigma. Heptyl iodide was obtained from Alfa Aesar. The solvents were dried and purified, when necessary, by appropriate standard procedures. Butyl methanesulfonate was synthesized following previously reported protocols [44,45]. 1,12-Diazido-4,9-dioxadodecan-2,11-diol (1) [46], 5 [47], 6 [48] and P1T [49] were synthesized following the procedures previously described. Vancomycin-resistant *Enterococcus facecium* (VRE), methicillin-resistant *Staphylococcus aureus* (MRSA), carbapenem-resistant *Klebsiella pneumoniae*, multidrug-resistant *Pseudomonas aeruginosa*, and *Enterobacter* spp. were obtained from Hospital Virgen de Valme (Sevilla, Spain). *Escherichia coli* CECT 101 was purchased from Colección Española de Cultivos Tipo (Valencia, Spain). Mueller-Hinton Broth (MHB) and Tryptic Soy Agar (TSA) were obtained from Scharlab (Barcelona, Spain). Bovine fetal serum, Dulbecco’s Modified Eagle’s Medium, antibiotics, *N*-(2-Hydroxyethyl)piperazine-*N′*-(2-ethanesulfonic acid) (HEPES), and trypsin were acquired from Thermo Fisher Scientific (Massachusetts, USA). Fresh human blood was obtained from a healthy volunteer. Human gingival fibroblasts were purchased from Innoprot (Vizcaya, Spain).

### 2.2. Measurements

Thin-layer chromatography (TLC) was performed on Silica Gel 60 F254 (E. Merck) with detection by UV light or charring with H_2_SO_4_ or phosphomolybdic acid. Flash column chromatography was performed using E. Merck Silica Gel 60 (230–400 mesh). Fourier transform infrared (FTIR) spectra were recorded on a Bruker Invenio-X equipped with an ATR spectrometer in the wavenumber range from 600 to 4000 cm^−1^ in the CITIUS of Universidad de Sevilla. ^1^H and ^13^C NMR spectra were recorded with a Bruker Avance Neo 500 MHz spectrometer in the CITIUS of the Universidad de Sevilla. Chemical shifts are reported as parts per million downfield from tetramethylsilane. COSY and HETCOR pulse sequences were used to record two-dimensional ^1^H–^1^H homonuclear and ^13^C–^1^H heteronuclear shift correlation spectra, respectively. Bruker Topspin software (version 3.1) was used for data acquisition and analyses. The thermal behavior of the polymers was examined by differential scanning calorimetry (DSC) using a TA DSC Q200 Instrument, calibrated with indium. Samples of about 2–3 mg were heated at a rate of 10 °C/min under a nitrogen flow rate of 20 mL/min and cooled to −35 °C. Thermogravimetric analyses (TGA) were carried out by a SDT Q600 TA instrument at a heating rate of 10 °C/min under a nitrogen flow of 100 mL/min, and the temperature range was from room temperature to 600 °C. A Waters apparatus equipped with a Waters 2414 refractive-index detector and two μStyragel HR columns (7.8 mm × 300 mm) linked in series, thermostatted at 60 °C, were used for Gel permeation chromatography (GPC) analyses. *N*, *N*-dimethylformamide containing 0.5 mg/mL LiBr was used as the mobile phase, at a flow rate of 1.0 mL min^−1^. Calibration was performed using polystyrene samples of narrow molecular-weight distribution. The total content of copper in the polymer samples was determined at *m*/*z* 63 with an Agilent 8800 inductively coupled plasma mass spectrometer (ICP-MS/MS) from Agilent technologies in the CITIUS of the Universidad de Sevilla with rhodium-103 as an internal standard. UV-vis absorbance was measured using a Bio Tek Sinergy HT plates reader (Winooski, VT, USA).

### 2.3. Monomer Synthesis

#### 2.3.1. Preparation of Monomer 2

Compound 1 (0.49 g, 1.7 mmol) was dissolved in DMSO (5.0 mL), and finely comminuted KOH (0.39 g, 7.0 mmol) was added to the solution under continuous agitation. Next, methyl iodide (0.57 g, 4.0 mmol) was added dropwise, and the reaction mixture was stirred for 24 h at room temperature. Then, water (7.5 mL) was added, and extractions were performed with dichloromethane (3 × 20 mL). The organic phase was dried over Na_2_SO_4_, filtered, and the solvent was removed under reduced pressure. The residue was purified by flash column chromatography using hexane-acetone (3:1) as eluent to give 2 as pale-yellow oil (0.48 g, 90%). IR: ν_max_ 2094 (N_3_), 1114 cm^−1^ (C-O); NMR data (300 MHz, CDCl_3_): ^1^H, δ 3.52–3.42 (m, 10H, H-2, 3, 4), 3.47 (s, 6H, H-6), 3.24–3.28 (m, 4H, H-1), 1.63 (m, 4H, H-5); ^13^C, δ 79.31 (C-2), 71.39, 69.74 (C-3, 4), 57.91 (C-6), 51.54 (C-1), 26.27 (C-5). HRMS: *m*/*z* 339.1744 (calcd. for [M+Na]^+^: 339.1751).

#### 2.3.2. Preparation of Monomer 3

To a mixture of compound 1 (0.35 g, 1.2 mmol), finely comminuted KOH (0.34 g, 6.1 mmol), tetrabutylammonium bromide (0.16 mg, 0.5 mmol), and H_2_O (50.0 µL), butyl methanesulfonate (1.52 g, 10.0 mmol) in toluene (1.0 mL) was added. The reaction mixture was refluxed at 120 °C for 24 h. Then dichloromethane (5 mL) was added, the mixture was filtered, and the solvents were removed under vacuum. Flash column chromatography was performed using a gradient of hexane-acetone as eluent (from 10:1 to 5:1). The fractions were collected, and solvents were removed to give 3 as a pale-yellow oil (0.20 g, 43%). IR: ν_max_ 2095 (N_3_), 1112 cm^−1^ (C-O); NMR data (300 MHz, CDCl_3_): ^1^H, δ 3.65–3.39 (m, 14H, H-2, 3, 4, 6), 3.39–3.22 (m, 4H, H-1), 1.68–1.50 (m, 8H, H-5, 7), 1.46–1.31 (m, 4H, H-8), 0.92 (t, 6H, H-9); ^13^C, δ 77.87 (C-2), 71.33, 70.28, 70.14 (C-3, 4, 6), 52.03 (C-1), 32.07 (C-7), 26.29 (C-5), 19.18 (C-8), 13.82 (C-9). HRMS: *m*/*z* 423.2689 (calcd. for [M+Na]^+^: 423.2690).

#### 2.3.3. Preparation of Monomer 4

Compound 1 (1.0 g, 3.5 mmol) was dissolved in DMSO (10.0 mL), and finely comminuted KOH (1.0 g, 18.0 mmol) was added to the solution under continuous agitation. Next, heptyl iodide (1.82 g, 8.0 mmol) was added dropwise to the mixture and the reaction mixture was stirred at room temperature overnight. Then H_2_O (15.0 mL) was added and extracted with dichloromethane (3 × 20 mL). The organic phase was dried over Na_2_SO_4_, filtered, and the solvent removed under vacuum. Undesired products were removed by flash column chromatography using hexane-acetone (5:1) as eluent. Compound 4 was obtained as an oil (1.46 g, 85%). IR: ν_max_ 2097 (N_3_), 1114 cm^−1^ (C-O); NMR data (300 MHz, CDCl_3_): ^1^H, δ 3.60–3.50 (m, 6H, H-2, 6), 3.50–3.39 (m, 8H, H-3, 4), 3.39–3.25 (m, 4H, H-1), 1.68–1.49 (m, 8H, H-5, 7), 1.42–1.20 (m, 16H, H-8, 9, 10, 11), 0.88 (t, 6H, H-12); ^13^C, δ 77.86 (C-2), 71.34, 70.14 (C-3, 4), 70.63 (C-6), 52.03 (C-1), 31.79, 29.09, 25.97, 22.59 (C-8, 9, 10, 11), 30.01 (C-7), 26.30 (C-5), 14.05 (C-12). HRMS: *m*/*z* 507.3627 (calcd. for [M+Na]^+^: 507.3629).

### 2.4. Polymer Synthesis

#### 2.4.1. Catalytic Method

Stoichiometric amounts of the corresponding bis-azide and bis-alkyne monomers were mixed in a flask and dissolved in *tert*-butanol (2 mL) under argon atmosphere. CuSO_4_·5H_2_O (5%) and sodium ascorbate (10%) were added to the solution, and then deionized water was added dropwise until light turbidity was observed. The mixture was stirred while heating at 50 °C. After 24 h, dichloromethane (5 mL) was added, and the reaction mixture was washed with deionized water (3 × 10 mL) and a saturated solution of NaCl in water (3 × 10 mL). The organic phase was separated, evaporated until dryness, dissolved in a minimum amount of dichloromethane, and precipitated in hexane. The isolated yields and their infrared and NMR spectroscopy data are listed below.

#### 2.4.2. Thermal Method

Stoichiometric amounts of the corresponding bis-azide and bis-alkyne monomers were mixed in a flask and the mixture was stirred and heated at 75 °C under argon atmosphere until the bis-alkyne monomer was completely melted. The temperature was then increased to 100 °C. After 24 h of heating, a minimum amount of dichloromethane was added to dissolve the residue and the solution was precipitated in hexane. The isolated yields and their infrared and NMR spectroscopy data are listed below.

P2C. Yield: 100% IR: ν_max_ 3333 (NH), 1671 (CO), 1517 cm^−1^ (amide II); NMR data (500 MHz, CDCl_3_): ^1^H, δ 8.15 (d, 2H, H-8), 7.67, 7.44 (bs, 2H, NH), 4.65 (bd, 2H, J_1a,1b_ 13.6 Hz, H-1a), 4.44 (dd, 2H, J_1b,2_ 7.1 Hz, J_1a,1b_ 13.6 Hz, H-1b), 3.69 (m, 2H, H-2), 3.58 (m, 4H, H-3), 3.55–3.25 (m, 16H, H-4, b, c, d), 3.55–3.25 (m, 4H, H-a), 3.34 (s, 6H, H-6), 1.63 (m, 4H, H-5), 1.43 (s, 27H, H-9); ^13^C, δ 160.50, 155.30 (CO), 143.06 (C-7), 126.60 (C-8), 80.21 (C-10), 78.53 (C-2), 71.53 (C-3), 68.82 (C-4), 58.02 (C-6), 51.67 (C-1), 46.86–44.72 (C-b, c, d), 38.12 (C-a), 28.33 (C-9), 26.29 (C-5).

P2T. Yield: 85% IR: ν_max_ 3330 (NH), 1669 (CO), 1520 cm^−1^ (amide II); NMR data (500 MHz, CDCl_3_): ^1^H, δ 8.15, 7.96 (d, H-8,8′), 7.65, 7.42 (bs, NH), 5.01–4.79 (m, H-1′), 4.65 (bd, J_1a,1b_ 13.6 Hz, H-1a), 4.44 (dd, 2H, J_1b,2_ 7.2 Hz, J_1a,1b_ 13.6 Hz, H-1b), 3.85 (m, H-2′), 3.69 (m, H-2), 3.65–3.20 (m, H-3, 3′, 4, 4′, a, a′, b-d, b′-d′), 3.34 (s, H-6, 6′), 1.66 (m, H-5, 5′), 1.43 (s, H-9, 9′); ^13^C, δ 160.50, 158.24, 155.50 (CO), 143.03, 131.49 (C-7, 7′), 133.91, 126.65 (C-8, 8′), 80.36–80.24 (C-10, 10′), 79.16 (C-2′), 78.52 (C-2), 71.59 (C-3′), 71.53 (C-3), 70.23 (C-4′), 68.81 (C-4), 58.29 (C-6′), 58.01 (C-6), 51.68 (C-1), 50.36 (C-1′), 46.80–44.72 (C-b, c, d, b′, c′, d′), 38.11 (C-a, a′), 28.44 (C-9′), 28.33 (C-9), 26.28 (C-5), 26.26 (C-5′).

P3T. Yield: 80% IR: ν_max_ 3335 (NH), 1674 (CO), 1572 cm^−1^ (amide II); NMR data (500 MHz, CDCl_3_): ^1^H, δ 8.13, 7.96 (d, H-11,11′), 7.63, 7.49 (bs, NH), 4.97–4.80 (m, H-1′), 4.64 (bd, H-1a), 4.42 (m, H-1b), 3.93 (m, H-2′), 3.78 (m, H-2), 3.68–3.20 (m, H-3, 3′, 4, 4′, 6, 6′, a, a′, b-d, b′-d′), 1.65 (m, H-5, 5′), 1.60–1.40 (m, H-7, 7′), 1.44 (s, H-12, 12′), 1.40–1.16 (m, H-8, 8′), 0.94–0.79 (t, H-9, 9′); ^13^C, δ 160.39, 158.26, 155.21 (CO), 142.94, 131.58 (C-10, 10′), 133.89, 126.60 (C-11, 11′), 80.32 (C-13, 13′), 77.66 (C-2′), 77.02 (C-2), 71.49, 71.33, 70.47, 70.32, 70.12, 69.35 (C-3, 4, 3′, 4′, 6, 6′), 52.10 (C-1), 50.90 (C-1′), 47.00–44.00 (C-b, c, d, b′, c′, d′), 38.08 (C-a, a′), 32.05, 31.82 (C-7, 7′), 28.43, 28.33 (C-12, 12′), 26.29, 26.35 (C-5, 5′), 19.10, 18.96 (C-8, 8′), 13.80, 13.74 (C-9, 9′).

P4C. Yield: 95%. IR: ν_max_ 3318 (NH), 1683 (CO), 1572 cm^−1^ (amide II); NMR data (500 MHz, CDCl_3_): ^1^H, δ 8.12 (s, 2H, H-14), 7.64, 7.41 (bs, 2H, NH), 4.63 (bd, 2H, H-1a), 4.22 (dd, 2H, J_1b,2_ 7.3 Hz, J_1a,1b_ 13.6 Hz, H-1b), 3.77 (m, 2H, H-2), 3.58 (m, 4H, H-a), 3.53–3.25 (m, 24H, H-3, 4, 6, b, c, d), 1.65 (m, 4H, H-5), 1.46 (m, 4H, H-7), 1.44 (s, 27H, H-15), 1.33–1.17 (m, 16H, H-8, 9, 10, 11), 0.87 (t, 6H, H-12); ^13^C, δ 160.43, 155.30 (CO), 142.99 (C-13), 126.55 (C-14), 80.18 (C-16), 77.10 (C-2), 71.50 (C-3), 70.70 (C-6), 69.38 (C-4), 52.20 (C-1), 46.30, 45.30 (C-b, c, d), 38.10 (C-a), 31.80, 28.90, 25.70, 22.70 (C-8, 9, 10, 11), 29.80 (C-7), 28.30 (C-15), 26.30 (C-5), 14.20 (C-12).

P4T. Yield: 100% IR: ν_max_ 3320 (NH), 1680 (CO), 1570 cm^−1^ (amide II); NMR data (500 MHz, CDCl_3_): ^1^H, δ 8.12, 7.95 (d, H-14, 14′), 7.64, 7.41 (bs, NH), 4.98–4.79 (m, H-1′), 4.64 (bd, H-1a), 4.42 (m, H-1b), 3.93 (m, H-2′), 3.78 (m, H-2), 3.70–3.20 (m, H-3, 3′, 4, 4′, 6, 6′), 3.65–3.54 (m, H-a, a′), 3.52–3.25 (m, H-b, c, d, b′, c′, d′), 1.65 (m, H-5, 5′), 1.46 (m, H-7, 7′), 1.33–1.17 (m, H-8, 9, 10, 11, 8′, 9′, 10′, 11′), 1.44 (s, H-15, 15′), 0.87 (t, H-12, 12′); ^13^C, δ 160.38, 158.24, 155.42 (CO), 142.99, 131.59 (C-13, 13′), 133.91, 126.61 (C-14, 14′), 80.18 (C-16, 16′), 77.70 (C-2′), 77.10 (C-2), 71.49, 71.33, 70.85, 70.70, 69.38 (C-3, 4, 3′, 4′, 6, 6′), 52.10 (C-1), 50.80 (C-1′), 47.00–44.50 (C-b, c, d, b′, c′, d′), 38.10 (C-a, a′), 31.80, 31.73, 29.05, 29.00, 25.88, 25.77, 22.70 (C-8, 9, 10, 11, 8′, 9′, 10′, 11′), 29.81 (C-7, 7′), 28.43, 28.33 (C-15, 15′), 26.40–26.25 (C-5, 5′), 14.20 (C-12, 12′).

P5C. Yield: 100%. IR: ν_max_ 3319 (NH), 1667 (CO), 1571 cm^−1^ (amide II); NMR data (500 MHz, CDCl_3_): ^1^H, δ 8.26 (bs, 2H, H-12), 7.64, 7.41 (bs, 2H, NH), 4.67 (dd, 2H, J_1a,2_ 2.0 Hz, H-1a), 4.45 (dd, 2H, J_1b,2_ 7.0 Hz, J_1a,1b_ 14.0 Hz, H-1b), 3.87 (m, 2H, H-2), 3.80–3.24 (m, 40H, H-3, 4, 6, 7, 8, 9, a, b, c, d), 3.36 (s, 6H, H-10), 1.64 (m, 4H, H-5), 1.44 (s, 27H, H-13); ^13^C, δ 160.49, 155.33 (CO), 143.00 (C-11), 126.86 (C-12), 80.20 (C-14), 77.43 (C-2), 71.87, 71.44, 70.70, 70.53, 69.75, 69.49 (C-3, 4, 6, 7, 8, 9), 58.97 (C-10), 51.72 (C-1), 46.40, 45.05 (C-b, c, d), 38.09 (C-a), 28.43, 28.33 (C-13), 26.29 (C-5).

P5T. Yield: 95%. IR: ν_max_ 3320 (NH), 1668 (CO), 1570 cm^−1^ (amide II); NMR data (500 MHz, CDCl_3_): ^1^H, δ 8.26, 7.91 (s, H-12, 12′), 7.63, 7.40 (bs, NH), 5.00–4.75 (m, H-1′), 4.67 (bd, H-1a), 4.45 (dd, H-1b), 3.96 (m, H-2′), 3.88 (m, H-2), 3.82–3.10 (m, H-3, 3′, 4, 4′, 6, 6′, 7, 7′, 8, 8′, 9, 9′, a, a′, b, b′, c, c′, d, d′), 3.36 (s, H-10, 10′), 1.63 (m, H-5, 5′), 1.44 (s, H-13, 13′); ^13^C, δ 160.50, 158.26, 155.33 (CO), 143.04, 131.68 (C-11, 11′), 133.96, 126.81 (C-12′, 12), 80.26 (C-14, 14′), 78.17 (C-2′), 77.51 (C-2), 72.00–69.40 (C-3, 4, 3′, 4′, 6, 6′, 7, 7′, 8, 8′, 9, 9′), 58.95 (C-10, 10′), 51.71 (C-1), 50.73 (C-1′), 46.50, 45.31 (C-b, c, d, b′, c′, d′), 38.02 (C-a, a′), 28.43, 28.33 (C-13, 13′), 26.29 (C-5, 5′).

#### 2.4.3. Removal of the N-Boc Protecting Groups of Polytriazoles

The selected polytriazole (0.4 g) was dissolved in dry dioxane (3.0 mL) and treated with 4N HCl in dioxane (10.0 mL) and stirred for about 3 h at room temperature to remove the *N*-Boc protecting groups. The reaction mixture was filtered, and the isolated solid was washed with ether and dried in vacuum to obtain the compound as the amine hydrochloride derivative in very high or quantitative yield. The isolated yields and their infrared and NMR spectroscopy data are listed below.

P1TD. Yield: 75%; IR: ν_max_ 3315 (OH), 2685 (broad,^+^NH_2_), 1655 (CO), 1572 cm^−1^ (amide II); NMR data (500 MHz, D_2_O): ^1^H, δ 8.40, 8.18 (s, H-7, 7′), 4.81 (dd, H-1′a), 4.73 (dd, H-1′b), 4.60 (dd, H-1a), 4.48 (dd, H-1b), 4.22 (m, H-2, H-2′), 3.74 (t, H-a), 3.70 (t, H-a′), 3.60–3.42 (m, H-3, H-3′, H-4, H-4′, H-c-d, H-c′-d′), 3.39 (t, H-b), 3.37 (m, H-b′), 1.57 (bs, H-5, H-5′); ^13^C, δ 163.00, 160.19 (CO), 141.55 (C-6), 134.80 (C-7′), 131.63 (C-6′), 127.97 (C-7), 71.49 (C-3′), 71.24, 71.20 (C-3, C-4, C-4′), 68.90 (C-2′), 68.43 (C-2), 53.28 (C-1), 52.53 (C-1′), 48.10 (C-b), 47.70 (C-b′), 43.58, 43.54, 43.23, 43.14 (C-c-d, C-c′-d′), 36.02 (C-a′), 35.77 (C-a), 25.29 (C-5, C-5′).

P2CD. Yield: 98%. IR: ν_max_ 3321 (NH), 2800–2250 (broad, ^+^NH_2_), 1656 (CO), 1573 cm^−1^ (amide II); NMR data (500 MHz, D_2_O): ^1^H, δ 8.42 (bs, 2H, H-8), 4.77–4.67 (dd, 2H, J_1a,2_ 3.9 Hz, H-1a), 4.58 (dd, 2H, J_1b,2_ 6.6 Hz, J_1a,1b_ 14.6 Hz, H-1b), 3.91 (m, 2H, H-2), 3.77 (t, 4H, J_a,b_ 5.5 Hz, H-a), 3.64 (dd, 2H, J_4a,5_ 4.20 Hz, J_4a,4b_ 11.20 Hz, H-4a), 3.54 (m, 4H, H-3), 3.46 (dd, 2H, J_4b,5_ 5.3 Hz, H-4b), 3.57–3.52 (m, 8H, H-c, d), 3.41 (t, 4H, H-b), 3.33 (s, 6H, H-6), 1.60 (m, 4H, H-5); ^13^C, δ 163.01 (CO), 141.69 (C-7), 128.06 (C-8), 77.91 (C-2), 71.27 (C-3), 68.74 (C-4), 57.38 (C-6), 50.84 (C-1), 48.07 (C-b), 43.16, 43.54 (C-c, d), 35.74 (C-a), 25.28 (C-5).

P2TD. Yield: 100%. IR: ν_max_ 3324 (NH), 2800–2250 (broad, ^+^NH_2_), 1660 (CO), 1572 cm^−1^ (amide II); NMR data (500 MHz, D_2_O): ^1^H, δ 8.42, 8.18 (bs, H-8, 8′), 4.80 (m, H-1′), 4.77–4.61 (H-1a), 4.54 (dd, J_1b,2_ 6.7 Hz, J_1a,1b_ 14.6 Hz, H-1b), 3.95–3.79 (m, H-2, 2′), 3.73 (t, J_a,b_ 5.5 Hz, H-a, a′), 3.65–3.30 (m, H-3, 3′, 4, 4′, c, c′, d, d′), 3.37 (t, H-b, b′), 3.30, 3.20 (s, H-6, 6′), 1.60 (m, H-5, 5′); ^13^C, δ 163.01, 160.26 (CO), 141.62 (C-7), 134.78 (C-7′), 131.64 (C-8′), 128.00 (C-8), 78.55 (C-2′), 77.88 (C-2), 71.24 (C-3, 3′), 68.70 (C-4, 4′), 57.60 (C-6′), 57.32 (C-6), 50.79 (C-1), 50.11 (C-1′), 48.04 (C-b), 47.55 (C-b′), 43.48, 43.17, 43.05 (C-c, c′, d, d′), 35.92 (C-a′), 35.71 (C-a), 25.20 (C-5, 5′).

P3TD. Yield: 100%. IR: ν_max_ 3302 (NH), 2800–2250 (broad, ^+^NH_2_), 1654 (CO), 1573 cm^−1^ (amide II); NMR data (500 MHz, D_2_O): ^1^H, δ 8.42, 8.20 (s, H-11,11′), 4.83 (bd, H-1′), 4.69 (H-1a), 4.53 (dd, J_1b,2_ 7.8 Hz, J_1a,1b_ 14.6 Hz, H-1b), 3.98 (m, H-2, 2′), 3.81–3.69 (m, H-a, a′), 3.68–3.20 (m, H-3, 3′, 4, 4′, 6, 6′), 3.59–3.45 (m, H-c, c′, d, d′), 3.39 (m, H-b, b′), 1.62 (m, H-5, 5′), 1.40–1.20 (m, H-7, 7′), 1.16–1.00 (m, H-8, 8′), 0.72 (t, H-9, 9′); ^13^C, δ 163.00, 160.14 (CO), 141.63, 131.58 (C-10, 10′), 134.91, 128.17 (C-11, 11′), 77.20 (C-2′), 76.67 (C-2), 71.31, 71.27, 70.55, 70.32, 69.75, 69.35 (C-3, 4, 3′, 4′, 6, 6′), 51.51 (C-1), 50.82 (C-1′), 48.00, 47.58 (C-b, b′), 43.55, 43.35, 43.22 (C-c, c′, d, d′), 35.89 (C-a′), 35.69 (C-a), 30.95 (C-7′), 30.90 (C-7), 25.33 (C-5, 5′), 18.45 (C-8), 18.37 (C-8′), 13.02 (C-9′), 12.98 (C-9).

P4CD. Yield: 95%. IR: ν_max_ 3321 (NH), 2800–2250 (broad, ^+^NH_2_), 1657 (CO), 1572 cm^−1^ (amide II); NMR data (500 MHz, D_2_O): ^1^H, δ 8.42 (s, 2H, H-14), 4.75–4.57 (m, 2H, H-1a), 4.50 (m, 2H, H-1b), 3.90 (m, 2H, H-2), 3.78 (m, 4H, H-a), 3.66–3.28 (m, 24H, H-3, 4, 6, b, c, d), 1.55 (m, 4H, H-5), 1.40 (m, 4H, H-7), 1.15 (m, 16H, H-8, 9, 10, 11), 0.76 (bt, 6H, H-12); ^13^C, δ 162.53 (CO), 141.68 (C-13), 127.91 (C-14), 77.03 (C-2), 71.23, 70.20, 69.97 (C-3, 4, 6), 51.34 (C-1), 48.05 (C-b), 43.83 (C-c, d), 35.70 (C-a), 31.81, 29.03, 25.86, 22.50 (C-8, 9, 10, 11), 29.70 (C-7), 25.89 (C-5), 13.83 (C-12).

P4TD Yield: 100%. IR: ν_max_ 3319 (NH), 2800–2250 (broad, ^+^NH_2_), 1657 (CO), 1574 cm^−1^ (amide II); NMR data (500 MHz, D_2_O): ^1^H, δ 8.39, 8.22 (s, H-14, 14′), 4.81–4.65 (m, H-1′), 4.54 (m, H-1), 3.92 (m, H-2′), 3.86 (m, H-2), 3.80–3.63 (m, H-a, a′), 3.50–3.23 (m, H-3, 3′, 4, 4′, 6, 6′), 3.56 (m, H-c, d, c′, d′), 3.40 (m, H-b, b′), 1.52 (m, H-5, 5′), 1.38, 1.32 (m, H-7, 7′), 1.13 (m, H-8, 9, 10, 11, 8′, 9′, 10′, 11′), 0.75 (bt, H-12, 12′); ^13^C, δ 162.46, 159.56 (CO), 141.63, 131.10 (C-13, 13′), 134.82, 127.84 (C-14, 14′), 77.53 (C-2′), 76.95 (C-2), 71.32–69.64 (C-3, 4, 3′, 4′, 6, 6′), 51.31 (C-1), 50.94 (C-1′), 48.02, 47.61 (C-b, b′), 43.90–43.38 (C-c, c′, d, d′), 35.85, 35.64 (C-a, a′), 31.80, 29.03, 25.85, 25.50 (C-8, 9, 10, 11, 8′, 9′, 10′, 11′), 29.70 (C-7, 7′), 25.90 (C-5, 5′), 13.83 (C-12, 12′).

P5CD. Yield: 100%. IR: ν_max_ 3317 (NH), 2800–2250 (broad, ^+^NH_2_), 1655 (CO), 1572 cm^−1^ (amide II); NMR data (500 MHz, D_2_O): ^1^H, δ 8.45 (bs, 2H, H-12), 4.80–4.65 (dd, 2H, J_1a,2_ 3.6 Hz, H-1a), 4.58 (dd, 2H, J_1b,2_ 7.3 Hz, J_1a,1b_ 14.6 Hz, H-1b), 4.05 (m, 2H, H-2), 3.76–3.48 (m, 24H, H-3, 4, 6, 7, 8, 9), 3.77 (t, 4H, Ha), 3.57–3.36 (m, 8H, H-c, d), 3.40 (t, 4H, H-b), 3.31 (s, 6H, H-10), 1.62 (m, 4H, H-5); ^13^C, δ 162.96 (CO), 141.58 (C-11), 128.16 (C-12), 76.92 (C-2), 71.33, 71.00, 69.77, 69.34, 69.12, 68.99 (C-3, 4, 6, 7, 8, 9), 58.08 (C-10), 51.36 (C-1), 47.97 (C-b), 43.53, 43.27 (C-c, d), 35.73 (C-a), 25.31 (C-5).

P5TD. Yield: 100%. IR: ν_max_ 3315 (NH), 2800–2250 (broad, ^+^NH_2_), 1654 (CO), 1571 cm^−1^ (amide II); NMR data (500 MHz, D_2_O): ^1^H, δ 8.45, 8.19 (s, H-12, 12′), 4.85 (bd, H-1′), 4.80–4.63 (H-1a), 4.57 (dd, H-1b), 4.04 (m, H-2, 2′), 3.82–3.29 (m, H-3, 3′, 4, 4′, 6, 6′, 7, 7′, 8, 8′, 9, 9′, a, a′, b, b′, c, c′, d, d′), 3.31, 3.30 (s, H-10, 10′), 1.61 (m, H-5, 5′); ^13^C, δ 162.96, 160.19 (CO), 141.57 (C-11), 131.11 (C-11′), 134.91 (C-12′), 128.15 (C-12), 77.52 (C-2′), 76.91 (C-2), 71.50–68.60 (C-3, 4, 3′, 4′, 6, 6′, 7, 7′, 8, 8′, 9, 9′), 58.08 (C-10, 10′), 51.35 (C-1), 50.68 (C-1′), 47.96, 47.56, 43.53, 43.28 (C-b, c, d, b′, c′, d′), 35.95 (C-a′), 35.73 (C-a), 25.30 (C-5, 5′).

### 2.5. Antibacterial Activity

The Minimum Inhibitory Concentration (MIC) was measured following the guidelines of the European Committee on Antimicrobial Susceptibility Testing by the microdilution method with minor modifications against clinical isolates of drug-resistant *Enterococcus faecium*, *Staphylococcus aureus*, *Klebsiella pneumoniae*, *Pseudomonas aeruginosa*, and *Enterobacter* spp. Stock solutions of each polymer were prepared in MHB, then serially diluted 2-fold in a 96-well plate from 1024 to 2 µg/mL. Colonies of each bacterial strain (3–4) were isolated, suspended in MHB and incubated at 37 °C under constant shaking. After 4 h, the turbidity of the bacterial suspensions was adjusted to 0.5 McFarland and diluted to reach a stock suspension of 1.5 × 10^6^ CFU/mL. Wells containing polymer solutions and growth control wells were inoculated with 100 µL of the bacterial suspension, and MHB (200 µL) was used as sterility control. The 96-well plate was incubated at 37 °C for 24 h, and MIC was defined as the lowest polymer concentration that inhibited the visible growth of the microorganism tested.

Minimum Bactericidal Concentration (MBC) was determined taking aliquots (10 µL) from the wells that did not have turbidity when MIC assays were performed and plating those aliquots in TSA plates. The TSA plates were then incubated at 37 °C for 24 h. MBC was defined as the lowest polymer concentration that prevented the growth of the microorganism tested after subculture onto polymer-free media. The experiments were carried out in triplicate.

### 2.6. Time-Kill Curves

Colonies of *Enterococcus faecium, Staphylococcus aureus*, *Klebsiella pneumoniae*, and *Pseudomonas aeruginosa* were isolated and cultured in MHB at 37 °C with constant agitation (180 rpm). After 4 h, the turbidity was adjusted to 0.5 McFarland and 100 µL of this stock suspension was added to a previously prepared solution of selected polymers in MHB at MIC concentration to reach a final concentration of around 5 × 10^4^ CFU/mL. The inoculated MHB without polymer was used as a growth control. Aliquots of the bacterial suspension were taken at 2, 4, and 8 h, serially diluted, and plated on TSA. After 24 h, colonies were counted. The experiment was repeated for triplicate readings.

### 2.7. Induced Resistance Test

Resistance studies were carried out following procedures [12,50] described previously. Colonies of *P. aeruginosa* and MRSA were cultured overnight in MHB at 37 °C under constant agitation. Bacterial suspensions were corrected to 0.5 McFarland, and their MIC values were calculated against P3TD, P2CD and P1TD, following the procedures described in Section 2.5. Cells growing at the highest polymer concentration (½ MIC) were harvested, centrifuged, and resuspended in MHB. Bacterial suspensions were corrected to 0.5 McFarland and incubated at 37 °C to determine the MIC value of bacterial cells exposed to these polycationic polymers. The experiment was carried out in 14 passages.

### 2.8. Hemolysis Assay

A stock solution of polymer was prepared in phosphate buffered saline (PBS, pH = 7.4) and serially diluted from 1024 to 2 µg/mL (100 µL). Fresh human blood was diluted in PBS and centrifuged at 700 G for 10 min, and the supernatant was discarded. Then, the human red blood cells (hRBC) pellet was resuspended in PBS and the procedure was repeated three times. PBS was added to reach 5% hematocrit and 100 µL of hRBC suspension was added to each well. The plate was incubated 1 h at 37 °C and centrifuged at 700 G for 10 min. Aliquots of 150 µL were transferred to a new 96-well plate, and the absorbance was measured at 540 nm. Wells containing only PBS were employed as negative control. Wells containing 2% Triton-X and hRBC were used as positive controls. The experiment was carried out three independent times, and the following equation was used to calculate the hemolytic activity:

$$\%\;hemolysis=\left(A_{540}^{polymer}-A_{540}^{negative}\right)/\left(A_{540}^{positive}-A_{540}^{negative}\right)$$

### 2.9. Cytotoxicity Assay

Cytotoxicity produced by antibacterial polymers in human gingival fibroblasts (HGnF) was measured using the widely applied MTT method based on the enzymatic-mediated reduction of 3-(4,5-dimethylthiazol-2-yl)-2,5-diphenyltetrazolium bromide to the colored water-insoluble crystal formazan. Cell culture was carried out in tissue flasks using Dulbecco’s modified Eagle medium (DMEM) supplemented with fetal bovine serum (FBS, 10%), L-glutamine and penicillin-streptomycin (1%). To perform cytotoxicity assays, HGnF cells were dissociated using trypsin, washed with supplemented DMEM and centrifuged. The pellet was resuspended in a complete medium; cells were counted, and a cell suspension was prepared (1.5 × 10^5^ cells/mL). 100 µL of this suspension were transferred to a 96-well plate and incubated at 37 °C with 5% CO_2_ for 24 h. The medium was then removed, and polymer dilutions were added in complete DMEM to the wells containing HGnF (1024 to 4 µg/mL). The medium without HGnF was used as a negative control, and HGnF with the polymer-free medium was used as a positive control. The plate was incubated for 24 h, and the medium was removed. Then 100 µL of fresh medium and 20 µL of MTT in PBS (5 mg/mL) were added to each well. The plate was incubated for 4 h at 37 °C in 5% CO_2_, the medium was removed and 150 µL of DMSO was added to each well. The absorbance was measured at 570 nm. The assays were performed in triplicate at three different times.

### 2.10. Cytoplasmic Membrane Depolarization Assay [51,52]

Colonies of *E. coli* CECT 101 were cultured in MHB. Mid-log phase bacteria were harvested and washed with buffer containing HEPES, glucose (5 mM) and KCl (100 mM). The turbidity of the bacterial suspension was corrected to optical density at 600 nm (OD_600_) 0.1, and DiSC_3_(5) (3,3′-dipropylthiadicarbocyanine iodide) was added to reach a final concentration of 1 µM; 190 µL was added to a 96-well black bottom plate. The plate was incubated for 35 min, and the fluorescence of DiSC_3_(5) was measured (E_x_ = 620 nm, E_m_ = 670 nm). Then, 10 µL of polymer in buffer solution was added to reach a final concentration of 0.5 mg/mL. Fluorescence was measured every 5 min. The assays were performed in triplicate at three different times.

### 2.11. Outer Membrane Permeabilization [51,52]

Colonies of *E. coli* CECT 101 were cultured in MHB. Mid-log phase bacteria were harvested and washed with HEPES-glucose 1:1 (5 mM). The turbidity of the bacterial suspension was corrected to OD_600_ 0.1, and NPN (*N*-phenyl-1-naphtylamine) was added to reach a final concentration of 10 µM; 190 µL were taken and placed on a 96-well black bottom plate. The plate was incubated for 5 min, and the fluorescence of NPN was measured (E_x_ = 350 nm, E_m_ = 420 nm); then 10 µL of polymer solution was added to reach a final concentration of 0.5 mg/mL. Fluorescence was measured every 5 min. The assays were performed in triplicate at three different times.

### 2.12. Inner Membrane Permeabilization [51]

Colonies of *E. coli* CECT 101 were cultured in MHB. Mid-log phase bacteria were harvested and washed with HEPES-glucose 1:1 (5 mM). The turbidity of the bacterial suspension was corrected to OD_600_ 0.1 and propidium iodide was added to reach a final concentration of 10 µM; 190 µL was added to a 96-well black bottom plate. The plate was incubated for 5 min, and the propidium iodide fluorescence was measured (E_x_ = 535 nm, E_m_ = 617 nm); then 10 µL of polymer solution was added to reach a final concentration of 0.5 mg/mL. Fluorescence was measured every 5 min. The assays were performed in triplicate at three different times.

### 2.13. Statistical Analysis

The results of the studies performed in triplicate are represented as mean ± standard deviation. The statistical significance of the results was determined by Student’s *t*-test. *p* < 0.05 was considered statistically significant. IBM SPSS statistics 26.0 (SPSS Inc., Chicago, IL, USA) software was used to perform the statistical analyses.

## 3. Results and Discussion

### 3.1. Synthesis and Characterization

We are interested in the preparation of polytriazoles because it constitutes a universal linking tool in polymer science, and the presence of triazole rings in the polymer chain could induce the development of biological activity by association with biological targets through hydrogen bonding and dipole interactions [53]. A large volume of research has been carried out on triazoles and their derivatives, demonstrating the pharmacological importance of this heterocyclic nucleus [54]. Here we describe the synthesis and characterization of seven new linear polytriazoles prepared by azide-alkyne click polymerization, in a solution catalyzed by copper (PnC) or by thermal cycloaddition (PnT).

**Figure 1:**
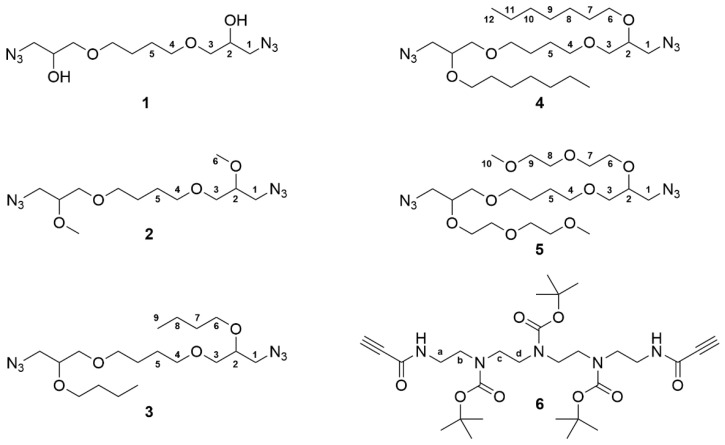
Structures of monomers.

**Figure 2:**
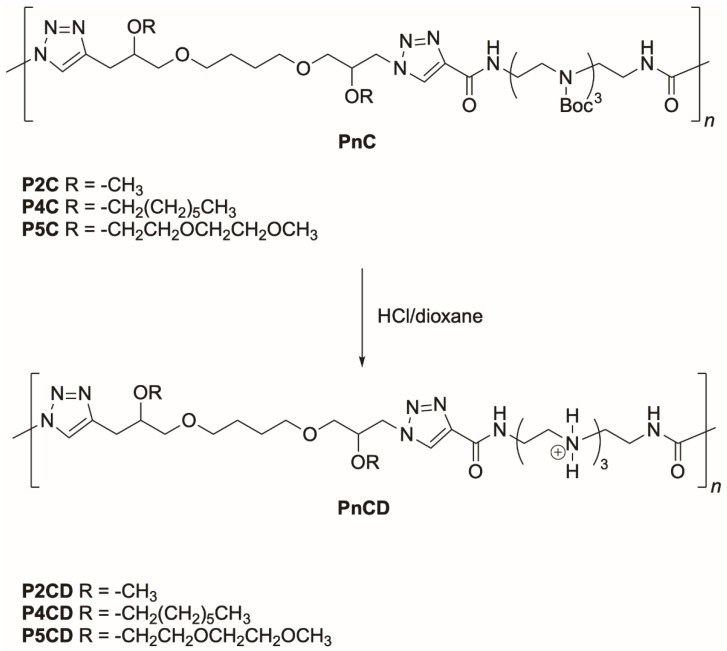
Structures of *N*-Boc protected polymers, PnC, prepared by CuAAC, and their corresponding deprotected polymers, PnCD.

Monomers represented in Figure 1 were used to obtain protected PnC and PnT polymers. Monomers 2–5 were prepared by alkylation reactions of the two secondary alcohol functions present in the structure of 1,12-diazido-4,9-dioxadodecan-2,11-diol (1). The syntheses of monomers 2 and 4 were approached using methyl and heptyl iodide, respectively, obtaining these compounds in high yield. On the other hand, monomers 3 and 5 were prepared by reacting the alcohol functions of monomer 1 with butyl and 2-(2-methoxyethoxy)ethyl mesylates, respectively, and the reaction yields were only modest. Monomers 2, 4, and 5 were polymerized with monomer 6, using the copper-catalyzed azide-alkyne cycloaddition reaction (CuAAC) to obtain the regiospecific polytriazoles P2C, P4C, and P5C (Figure 2).

**Figure 3:**
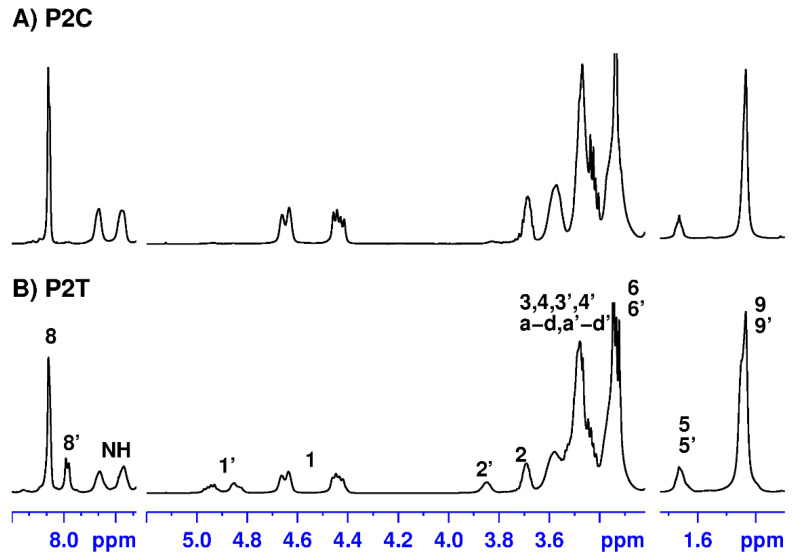
^1^H NMR spectra of polymers P2C and P2T recorded in deuterochloroform.

Furthermore, monomers 2–5 were also polymerized with bis-alkyne 6 by thermal cycloaddition reactions in the absence of solvent and catalyst. In this way, the PnT polymers were synthesized and obtained as a mixture of 1,4- and 1,5-disubstituted triazoles. Infrared spectroscopy and nuclear magnetic resonance data for all products described in this paper are shown in the Materials and Methods section. Infrared spectra show absorption bands for N-H stretching (3317–3335 cm^−1^), carbonyl group (1654–1683 cm^−1^), and N-H bending for amide II (1517–1573 cm^−1^). The ^1^H and ^13^C nuclear magnetic resonance data support the chemical constitution expected for these polymers that were isolated in high yield. As a representative example, Figure 3 shows the ^1^H NMR spectra of polytriazoles P2C and P2T, recorded in deuterochloroform, together with the assignments of the signals appearing in the P2T spectrum. The signal assignment was performed based on the information provided by the two-dimensional homonuclear and heteronuclear correlation spectra (shown in Supplementary materials Figures S1–S4).

The ^1^H NMR spectra of the prepared polymers clearly show the presence of the triazole rings, which must be formed during the click polymerization process. Thus, in the ^1^H NMR spectrum of the polymer P2C (Figure 3A), which is obtained by the catalytic procedure, a single signal was observed at 8.15 ppm that was assigned to the aromatic proton of the 1,4-triazole rings. On the other hand, in the spectrum of the P2T polymer (Figure 3B), obtained by thermal cycloaddition reactions, two signals centered at 8.15 and 7.96 ppm were observed, corresponding to the aromatic protons of the 1,4- and 1,5-triazole rings, which are formed by this procedure. In addition, in the spectrum of the P2T polymer, the splitting of some signals, such as H-1 and H-2, was observed due to the presence of the two regioisomeric triazole rings in the polymer chain. This fact is most clearly revealed in the ^13^C NMR data of the thermally obtained polymers, as detailed in the NMR spectroscopic data of the Experimental Section.

**Figure 4:**
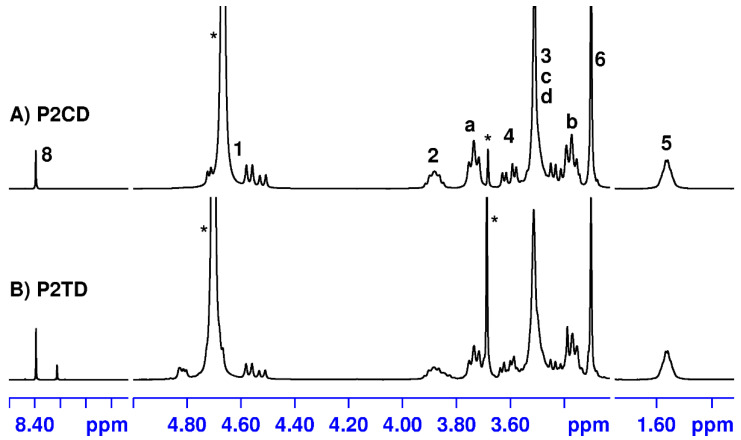
^1^H NMR spectra of the deprotected polymers P2CD and P2TD recorded in deuterium oxide. Signals marked with an asterisk correspond to solvents.

**Figure 5:**
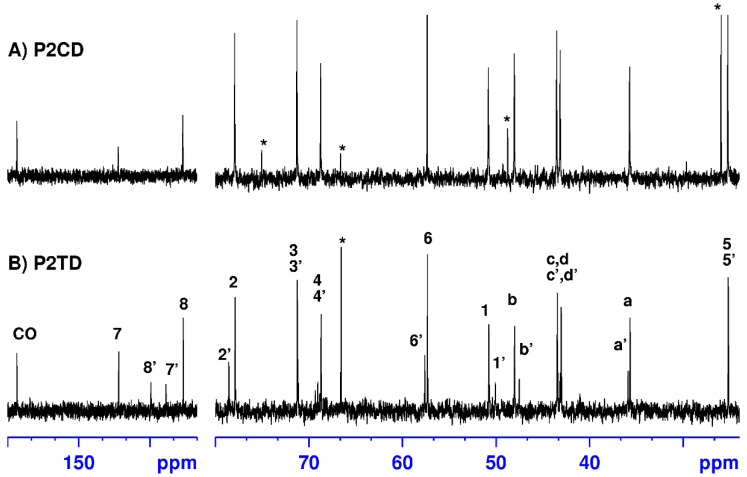
^13^C NMR spectra of the deprotected polymers P2CD and P2TD recorded in deuterium oxide. The P2TD spectrum shows duplication of some signals due to the presence of two regioisomeric triazole rings in the polymer chain. Signals marked with an asterisk correspond to solvents.

Elimination of *N*-Boc-protecting groups with HCl in dry dioxane affords the corresponding polycationic polymers in practically quantitative yields (Figure 2). ^1^H and ^13^C NMR spectra of the deprotected polymers P2CD and P2TD are shown in Figure 4 and Figure 5 together with the assignments of the signals that appear in the spectra, for illustration.

The signal corresponding to the resonance of the methyl groups of the *tert*-butyl carbamate functions, which appeared at 1.43 ppm (H-9, H-9′) in the protected polymer spectra (Figure 3), is no longer observed in the ^1^H NMR spectra of the deprotected polymers (Figure 4). Likewise, ^13^C NMR spectra (Figure 5) also support the removal of *N*-Boc protecting groups in the polymers. Thus, the signals that appeared at approximately 28, 80, and 155 ppm in the spectra of the protected polymers (see experimental section), and which corresponded to the *tert*-butyl and carbonyl groups of the carbamate functions, are no longer observed.

The presence of traces of the metallic catalyst in PnCD polycationic polymers was measured by ICP-MS analysis. As expected, the introduction of positive charges along the polymer backbone made these materials very readily soluble in water. However, in other protic solvents, such as methanol, they were only slightly soluble. The deprotected polymers, PnCD and PnTD, also readily dissolved in polar aprotic solvents such as DMSO and DMF but were insoluble in organic solvents such as acetone, dichloromethane, or ether.

### 3.2. Gel Permeation Chromatography

Table 1 shows the mass-averaged molar-mass (Mw) values for PnC and PnT protected polymers, which range from 43,000 to 269,000 g/mol. When the polymers obtained from the same monomers are compared, those obtained by the thermal cycloaddition reactions always have lower molar mass values than those obtained by the catalytic means. In the case of the deprotected polymers PnCD and PnTD, the molar mass values could not be determined because the final part of their chromatograms showed retention times close to the system signals and could not be integrated under the measurement conditions.

### 3.3. Thermal Analysis

**Figure 6:**
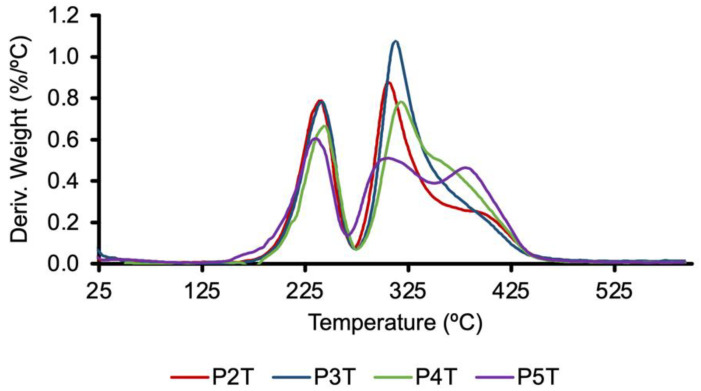
Derivative of TGA curves for polytriazoles: P2T, P3T, P4T, and P5T, measured at 10 °C/min.

The thermal behavior of the protected and unprotected polymers was studied by thermogravimetric analysis (TGA) and differential scanning calorimetry (DSC); the corresponding data are shown in Table 2. The thermal stabilities of polytriazoles were studied by following the mass loss of the sample as a function of temperature, using TGA. The thermal decomposition of protected polymers PnC and PnT, determined by TGA, begins, measured for 10% weight loss, around 217 °C. The decomposition processes took place in three stages, the first occurring around 230–240 °C, and the last is, in most cases, a shoulder of the second stage (Figure 6). The first thermal decomposition step can be attributed to the loss of the *tert*-butyl groups of the *N*-Boc protecting groups [55]; this decomposition step does not appear in the deprotected samples, and the mass loss corresponding to this first thermal decomposition is in good agreement with the value that should result according to the expected structure of the repeating unit of the polymer.

The deprotected polymers, PnCD and PnTD, have a higher decomposition temperature, measured at 10% weight loss, than the corresponding protected polymers. Thermal decomposition of these polymers occurs in two stages. Differential scanning calorimetry (DSC) provided the glass transition temperature values of the amorphous phase of the polymers (T_g_). T_g_ values range between 27 and 80 °C for protected polymers and between 39 and 112 °C for deprotected polymers. No melting endotherms were observed, suggesting that they exhibit essentially amorphous behavior.

In general, it is observed that the polymer samples obtained by the thermal process have lower T_g_ values than the polymers obtained by catalytic means, T_g_ (PnT) < T_g_ (PnC). The same is observed in unprotected polymers: T_g_ (PnTD) < T_g_ (PnCD). This behavior is probably related to the differences imposed by the main chain of the polymer, which is not regular in the case of the polymers obtained by the thermal route, as they present 1,4/1,5-disubstituted triazole units; in the case of the polymers obtained by catalytic means, their structure is regular, presenting only 1,4-disubstituted triazole units. The P1T and P1TD polymers have the highest T_g_ values between the protected and deprotected polymers, respectively (Table 2). The presence of the two alcohol functions in their repeating units would be responsible for these results, as hydrogen bonds can be established between the polymer chains. In the other polymers, a correlation can be observed between the T_g_ values presented by the polymer and the chain size of the alkyl radicals introduced by alkylation of the alcohol functions present in P1T. Thus, if we compare the protected polymers obtained by thermal treatment, PnT, or the polymers obtained by catalytic treatment, PnC, as the size of the alkyl chain (methyl, butyl, and heptyl) increases, the value of T_g_ progressively decreases. The same is observed if we use the deprotected polymers. The presence of diethylene glycol chains, instead of heptyl chains, in the repeating units of the P5T, P5C, P5TD and P5CD polymers causes these polymers to have the lowest T_g_ values. The introduction of short chains (methyl-heptyl) by alkylation of the alcohol functions of monomer **1** exerts a plasticizing effect on the thermal properties of the polymers obtained.

### 3.4. Antibacterial Activity

In order to evaluate the ability of the synthetized polymers to inhibit bacterial growth, antibacterial activity was tested against some pathogens in the ESKAPE group (*E. faecium, S. aureus, K. pneumoniae, P. aeruginosa,* and *Enterobacter* spp.). The MIC of polycationic polymers was investigated through broth microdilution assay. The results obtained (Table 3) against Gram-positive strains (*E. faecium* and MRSA) by unbranched (P1TD) and methylated branches polymers (P2TD and P2CD) were similar (16 µg/mL). When the length of the side chains was increased from one to four carbon atoms, P3TD improved its MIC (4 µg/mL) against the same strains. However, if the size of the side chains continues to increase, the MIC does not decrease but increases to higher values against all strains tested, as shown in the case of polymers P4CD and P4TD, which have heptylated branches.

Polymers P5TD and P5CD with hydrophilic side chains derived from diethylene glycol methyl ether (DEGME) were prepared. The P5TD polymer had MIC values of 256 µg/mL and 128 µg/mL against *E. faecium* and MRSA, respectively. In the case of the P5CD polymer, the MIC was somewhat lower, at 64 µg/mL, against both Gram-positive strains. *K. pneumoniae* presented high MIC values (> 1024 µg/mL) against polycationic polymers with hydrophilic branches (P5TD and P5CD). However, these values decreased significantly when methylated, butylated and unbranched polymers were used. The butylated polymer showed the lowest MIC (8 µg/mL). Table 3 shows that the P3TD polymer, which has butylated side chains, is the one with the highest antibacterial activity against bacterial species tested from the ESKAPE group, compared to the other polycationic polymers synthesized in this study.

Similarly, differences were found between the MIC values of thermal (non-regiospecific) and catalytic (regiospecific) polymers (Table 3). P2TD and P2CD had MICs of 256 and 64 µg/mL against *K. pneumoniae*, respectively; while in the case of *P. aeruginosa* the observed MIC values were lower, but in the same direction, that is, 128 and 32 µg/mL, respectively. In the case of the P4TD and P4CD pair, the same trend was observed against *Enterobacter* spp., in which the catalytic polymer (P4CD) had a somewhat lower MIC value than P4TD. Similar behavior was observed when *P. aeruginosa* was tested. The P4CD polymer presented a MIC of 128 µg/mL while the thermal polymer (P4TD) had values higher than 1024 µg/mL. The heptylated pair of polymers showed low antibacterial activity against Gram-negative strains and no activity against Gram-positive species. These results are in line with those of Oh et al., who prepared polymers with different side chains [56]. They found that, unlike polymers with shorter side chains, polymers with seven-atom side chains did not exhibit antibacterial activity. Finally, the polymer pair P5TD and P5CD, with hydrophilic side chains, also showed high MIC values against *E. faecium* and *S. aureus*, and in both cases, the P5CD polymer showed values somewhat lower than those of the P5TD polymer. In general, as seen for most cationic antimicrobial polymers, these polymers also showed better inhibition against Gram-positive bacteria.

The PnCD polytriazoles studied in this work were prepared by the catalytic procedure known as CuAAC, which carries the risk of using copper that can be toxic to mammalian cells. The amount of residual copper present in the final deprotected polymers can be easily and accurately determined by inductively coupled plasma mass spectrometry analysis. This technique showed that PnCD polymers had 0.006 g of copper per g of polymer as a maximum. This fact should not influence the results, since it is known [12,57] that a copper concentration of around 100 μM does not produce harmful effects.

### 3.5. Bactericidal Kinetics

**Figure 7:**
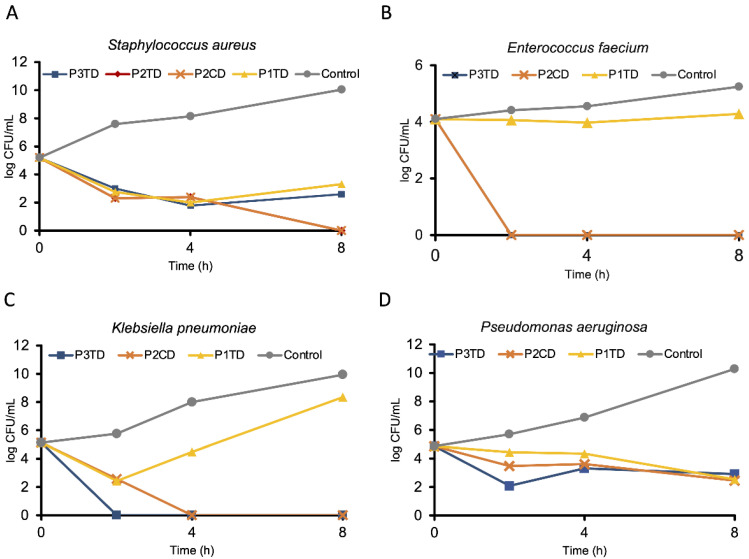
Concentration of viable cells given in log colony-forming units per milliliter plotted as a function of time. The polymer concentrations were used at their respective MIC values. (**A**) *Staphylococcus aureus* cells after incubation with P1TD, P2CD, P2TD, P3TD, and under control conditions. (**B**) *Enterococcus faecium* cells after incubation with P1TD, P2CD, P3TD, and under control conditions. (**C**) *Klebsiella pneumoniae* cells after incubation with P1TD, P2CD, P3TD, and under control conditions. (**D**) *Pseudomonas aeruginosa* cells after incubation with P1TD, P2CD, P3TD, and under control conditions. Control conditions implied no polymer added.

To determine whether the polymers have a bactericidal or bacteriostatic activity over time, kinetic studies were assessed. Polymers which showed low MIC values were selected to carry out in vitro killing kinetic studies against *K. pneumoniae*, *E. faecium*, MRSA, and *P. aeruginosa*. When polymers P1TD, P2TD, P2CD and P3TD were tested against MRSA (Figure 7A), all polymers showed bacteriostatic effects at 2 and 4 h, and P2CD and P2TD showed bactericidal effects, which is defined as the reduction of at least 3 log_10_ CFU/mL [58], after 8 h. Polymers P1TD, P2CD, and P3CD were tested against *E. faecium* (Figure 7B). P1TD did not show a significant reduction in survival, but P2CD and P3TD killed 99% of CFU and maintained their bactericidal effects during all experiments. Polycationic polymers P1TD, P2CD, and P3TD were tested against *K. pneumoniae* (Figure 7C). After 2 h, P1TD and P2CD showed bacteriostatic effects, while P3TD showed a bactericidal effect. This bactericidal effect was maintained during the experiment. After 4 h, P1TD showed a significant regrowth, and P2CD reached a bactericidal effect that lasted (99% killing). Finally, the polymers P1TD, P2CD and P4TD were tested against *P. aeruginosa* (Figure 7D), and all presented bacteriostatic effects at 2, 4, and 8 h.

### 3.6. Induced Resistance

Repeated or prolonged use of antimicrobial and anticancer drugs can cause bacteria to develop resistance [59,60]. Therefore, multiple drug-resistant strains have been isolated, such as MRSA, which are causing serious health problems due to the failure of clinical treatments. Antimicrobial polymers are often proposed as good candidates to combat bacteria because they do not usually induce resistance. However, it is important to study the susceptibility that bacteria may have to these materials, since in certain cases they also cause the appearance of resistance. We selected *P. aeruginosa* and MRSA as representative Gram-negative and -positive species to determine if their MICs increase after continuous exposure to sub-MIC concentrations of P1TD, P2CD and P3TD. To do this, they were exposed to the corresponding polymers in concentrations equal to half the MIC for 14 passages, and the ability of these antibacterial polymers to produce resistance was evaluated by checking their MIC values after each passage.

**Figure 8:**
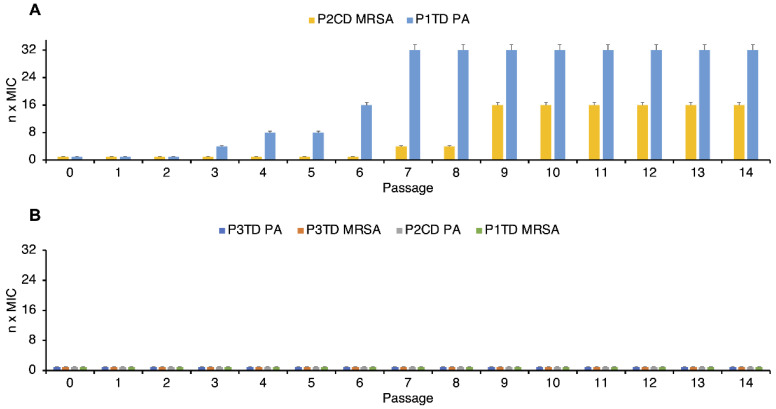
Propensity of *Staphylococcus aureus* and *Pseudomonas aeruginosa* (PA) to develop resistance to deprotected polytriazoles P1TD, P2CD and P3TD as a function of the number of passages: (**A**) Resistance appears; (**B**) No resistance appears. n MIC: n was the number by which the MIC value was multiplied in each passage. Standard deviation is plotted with error bars in the graphs.

Figure 8 shows the variation of the MIC values of the polymers studied as a function of the number of passages for *P. aeruginosa* and MRSA. As seen in Figure 8B, MICs of P3TD against tested strains did not suffer any variation after 14 passages. On the other hand, MIC of P2CD did not fluctuate against *P. aeruginosa*, but in the case of MRSA, MIC increased 4-fold after seven passages and 16-fold after nine passages (Figure 8A). MIC of P1TD did not change in the case of MRSA (Figure 8B). However, after three passages its MIC increased 4-fold for *P. aeruginosa*. P1TD MIC value was 32-fold higher after seven passages (Figure 8A).

### 3.7. Membrane Stability Studies

Three assays were performed to determine whether the selected polytriazoles interact with the bacterial membrane or not. We focused on a Gram-negative model microorganism of medical relevance, such as *E. coli*. Bacterial membrane depolarization was carried out using DiSC_3_(5) (3,3′-dipropylthiadicarbocyanine iodide), which is a cationic, fluorescent, and hydrophobic compound. DiSC_3_(5) produces an automatic quenching of its fluorescence when distributed on the surface of the hyperpolarized state of the cell membrane under the effect of KCl. When a polymer binds to the bacterial membrane, its membrane potential is disrupted, ion channels are generated, and unregulated ion transmembrane movements occur [61]. DiSC_3_(5) is released into the medium, producing an increase in the intensity of the fluorescence.

**Figure 9:**
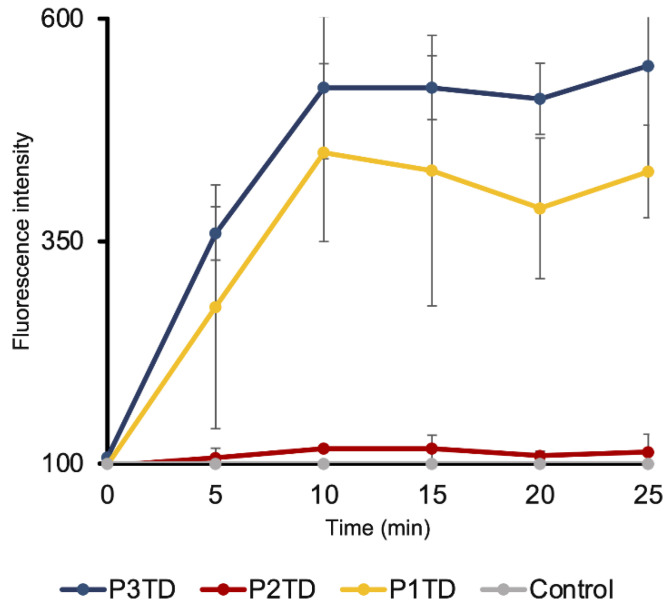
Cell membrane depolarization of *E. coli* by cationic polymers P1TD, P2TD and P3TD measured by the increase in DiSC_3_(5) fluorescence. Standard deviation is plotted with error bars in the graph. Polymer concentration was 0.5 mg/mL.

Correct electrical potential across the membrane is crucial, as it is a source of free energy (membrane transport, ATP synthesis and mobility). This potential is generally assumed to be homeostatic (pH, cell division and dynamic communications) [62]. Any disturbance in the polarization of the bacterial membrane could lead to dysfunctional membrane protein performance and subsequently cell death. To monitor the phenomenon of membrane depolarization, *E. coli* was incubated with polymers P2TD, P3TD, and P1TD, in HEPES-glucose containing DiSC_3_(5) 0.5 μM. The assay showed an increase of the detected fluorescence intensity upon the addition of the polymer, which was indicative of membrane depolarization. Figure 9 shows that in *E. coli*, polymers P3TD and P1TD induced a significant increase in DiSC_3_(5) fluorescence, while in the case of polymer P2TD the increase was not significant (Figure 9).

**Figure 10:**
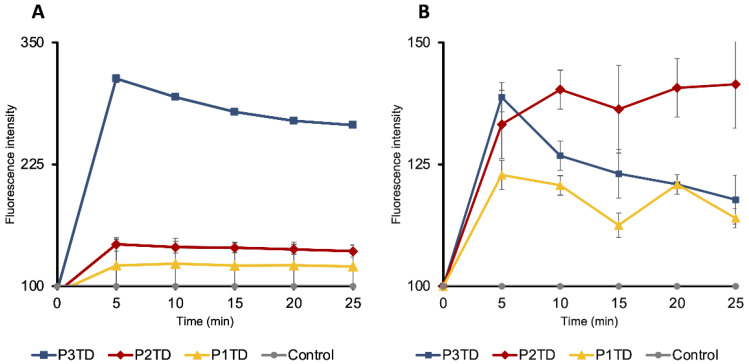
Cell membrane permeability of *E. coli* by cationic polymers P1TD, P2TD and P3TD: (**A**) Outer-membrane by the increase in NPN fluorescence; (**B**) Inner-membrane by the increase in PI fluorescence. Standard deviation is plotted with error bars in the graphs. Polymer concentration was 0.5 mg/mL.

In addition, the permeability of the inner and outer membranes was checked. The outer membrane acts as a permeability barrier that prevents hydrophobic substances from entering the cytosol. For this reason, NPN, a fluorescent marker, cannot pass through it and shows weak fluorescence in the buffered medium. However, when membrane integrity is compromised by the presence of a permeabilizing agent, NPN enters the phospholipid bilayer (hydrophobic environment), resulting in increased fluorescence. The addition of polymers P3TD, P2TD, or P1TD to *E. coli*, in the presence of NPN, produced a time-dependent increase in fluorescence emission (indicative of NPN uptake). The polymers bonded electrostatically to the cell surface, and this interaction led to an enhancement of cell permeability. We observed that the membrane permeation ability of polymers increased with an increase in the overall hydrophobicity of polytriazole (Figure 10A). Propidium iodide (PI) was used to determine the permeability of the inner membrane. Cells with damaged membranes take up this compound, which binds to nucleic acids, producing an increase in fluorescence. A similar trend is observed, with polytriazole **P3TD** being the one that initially produced a higher intensity of fluorescence. However, its value decreased over time, while in the other polymers it remained the same during the analyzed time (Figure 10B).

Polymers P3TD, P2TD and P1TD at 0.5 mg/mL showed depolarization and permeabilization of the cytoplasmic membrane when tested against *E. coli*, in the presence of DiSC_3_(5), NPN, and PI. Based on the results, as well as on the bibliography [63,64], one mechanism of antibacterial activity could be suggested. First, the cationic domains of polytriazoles possibly interact with the bacterial electrostatic net negative surface charge. Second, the hydrophobic domains might directly insert into the lipid core of the target membrane, leading to its disruption, which promotes cell death.

### 3.8. Hemolytic Activity

An initial way to evaluate the toxicity of new therapeutic agents to the human body is to check that they are not harmful to human red blood cells. hRBC lysis was evaluated spectrophotometrically by measuring the absorbance of the medium at 540 nm, which is directly proportional to the release of hemoglobin. To measure the selectivity that a polymer shows for bacteria against red blood cells, the HC50/MIC ratio is widely used, as it represents the polymer concentration that induces 50% of hemoglobin release from hRBC into the medium related to the MIC values (Table 4). Alkylation of the alcohol functions present in monomer **1**, and therefore the presence of alkyl chains in repeating units of the different polymers (Figure 2), generally caused a notable increase in hemolysis activity. Only polymers P5CD and P5TD, which carry diethylene glycol-derived radicals and therefore are hydrophilic in nature, were not hemolytic at all. However, the butylated polymer P3TD showed higher hemolytic activity than the heptylated pair of polymers P4TD and P4CD, which were expected to be more hemolytic due to their higher lipophilicity. In addition, although differences were observed between thermal and catalytic polymers, we did not find clear correlations. Under the conditions studied, the selectivity of the polymers tested by selected bacteria versus red blood cells is favorable for the P2CD, P4TD, P5CD, and P5TD polymers. The selectivity values in the case of the P2CD polymer were at least 64 for *Enterococcus faecium*, *Staphylococcus aureus* and *Enterobacter* spp., 32 for *Pseudomonas aeruginosa* and 16 for *Klebsiella pneumoniae*. In the case of the P4TD polymer, it presented a value of 16 for *Enterobacter* spp.

### 3.9. Cytotoxicity Assay

**Figure 11:**
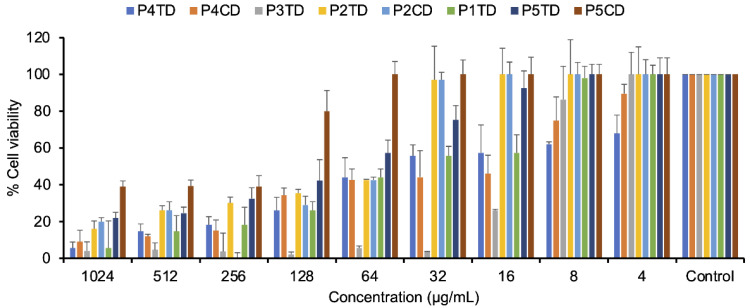
Cell viability of human gingival fibroblast cells was determined after 24 h of incubation with polymers P1TD, P2CD, P2TD, P3TD, P4CD, P4TD, P5CD, and P5TD, by the MTT assay. Standard deviation is plotted with error bars in the graph. Control conditions implied no polymer added.

The toxicity of the polymers was assessed using the MTT method. For this, human gingival fibroblast cells (HGnF) were used as representative mammalian cells, and the data corresponding to cell viability as a function of polymer concentration, after one day of incubation, is shown in Figure 11. The cytotoxicity and antimicrobial activity of the polymers depend on the ratio of hydrophobic to hydrophilic content. Therefore, the incorporation of hydrophilic fragments such as poly(ethylene glycol) [65,66] and sugars [67] can considerably improve the biocompatibility of antibacterial polymers. Although these modifications can be made without significantly reducing antibacterial activity, in our case an appreciable reduction in antibacterial activity was found when more hydrophilic side chains were introduced, such as those derived from diethylene glycol in P5CD and P5TD polymers. Polymers P5CD and P5TD had low antibacterial activity against *Enterococcus faecium* and *Staphylococcus aureus*. Furthermore, in the case of polymer P5CD, cellular viability was 100% at the MIC concentration and 80% at a concentration twice its MIC; while in the case of the polymer obtained under thermal conditions, P5TD, the viability values were lower, 60 and 40%, respectively. The polymers, P2CD and P4CD, obtained under catalytic conditions showed greater cytotoxicity than their corresponding thermal polymers. The P4TD and P4CD polymers showed low antibacterial activity against *Enterobacter* spp. At concentrations close to their MIC, cell viability values of around 40% were obtained. In the case of P2CD and P2TD, viabilities of 71% and 100%, respectively, were confirmed at the MIC concentration found for *E. faecium*, *S. aureus* and *Enterobacter* spp., and lower cell viabilities were found when concentrations corresponding to the MICs for *P. aeruginosa* (32 mg/mL) and *K. pneumoniae* (64 mg/mL) were used. The polymer that showed the greatest antibacterial activity was P3TD, which showed a cell survival of 100% at the concentration of its MIC (*E. faecium*, *S. aureus* and *Enterobacter* spp.), and 86% at a concentration twice its MIC (*K. pneumoniae and P. aeruginosa*), but greatly increased its cytotoxicity by increasing the concentration.

## 4. Conclusions

Several polytriazoles have been synthesized containing the same main chain but differing in their side chains. In this way, the hydrophobicity-hydrophilicity relationship in the antibacterial polymer can be modulated as a function of the length of the alkyl radical of the side chains or by their nature. In general, the resulting cationic polymers are antibacterial and active against different ESKAPE pathogens. Polymers carrying ethylene glycol (more hydrophilic) and heptyl (more lipophilic) side chains were found to have milder antibacterial activity. The P3TD polymer, which was prepared by a thermal cycloaddition reaction and has butylated side chains, was the one with the highest antibacterial activity against bacteria in the tested ESKAPE group, although it also had a high hemolytic activity. Methylated polymers (P2CD and P2TD) have a better balance between hydrophilicity and hydrophobicity, since they had good antibacterial properties and acceptable biocompatibility with hRBC and HGnF. The resistance that *P. aeruginosa*, selected as a Gram-negative strain, and MRSA, selected as a Gram-positive species, could develop against polymers P1TD, P2CD, and P3TD was studied. It is shown that neither *P. aeruginosa* nor MRSA increased their MICs to the polymeric antibacterial agents tested: P2CD and P3TD in the first case, and P3TD and P1TD in the second. Based on MIC values, the regiospecific polymers, PnCD, exhibited better antibacterial activity than their non-regiospecific analogs, PnTD. This effect may be due to the difference in regiospecificity or differences in molecular weight, since the polymers obtained by catalytic means had higher molecular weight values.

## Figures and Tables

**Table 1:** GPC ^1^ data of *N*-Boc-protected polytriazoles.

Polymer	Yield (%)	M_w_	M_w_/M_n_
P2C	100	269,200	2.0
P2T	85	139,800	2.2
P3T	80	43,000	1.3
P4C	95	136,400	1.3
P4T	100	78,800	1.6
P5C	100	155,300	1.9
P5T	95	102,000	1.6

^1^ Mass average molecular weight (Mw), number average molecular weight (Mn) in g/mol and dispersity (Mw/Mn) measured by GPC analysis in DMF/LiBr as mobile phase against polystyrene standards.

**Table 2:** Thermal analysis data of *N*-Boc-protected and deprotected polytriazoles.

Polymer	T_g_^1^ (°C)	T_10%_^2^ (°C)	T_dec_^3^ (°C)
P1T	80.0	219.0	235.0, 301.0
P2C	66.5	227.0	240.3, 300.0, 390.0 (s)
P2T	61.2	222.7	239.4, 306.0, 390.0 (s)
P3T	48.8	226.1	240.8, 312.5
P4C	38.2	232.1	244.1, 318.0, 354.0 (s)
P4T	31.9	232.6	243.5, 317.6, 354.1 (s)
P5C	28.6	227.0	241.0, 306.0, 380.0 (s)
P5T	27.2	217.5	234.4, 304.1, 380.8
P1TD	112.4	258.0	309.0, 338.0 (s)
P2CD	91.4	269.0	298.0, 358.0
P2TD	87.7	271.0	298.0, 353.0
P3TD	71.2	263.0	297.0, 371.0
P4CD	85.9	267.4	305.8, 360.9
P4TD	66.0	274.0	315.8, 359.8
P5CD	44.9	272.3	294.0, 368.0
P5TD	39.2	271.0	295.5, 367.5

^1^ Glass transition temperature taken as the inflection point of the second heating DSC traces recorded at 10 °C/min. ^2^ Temperature at which 10% weight loss was observed in TGA traces recorded at 10 °C/min. ^3^ Temperature for maximum degradation rate measured by TGA at 10 °C/min.

**Table 3:** Antibacterial activity of polycationic polymers MIC/MBC (μg/mL).

Polymer	*Enterococcus faecium*	*Staphylococcus aureus*	*Klebsiella pneumoniae*	*Pseudomonas aeruginosa*	*Enterobacter* spp.
P1TD	16/16	16/16	32/32	32/32	16/16
P2CD	16/32	16/16	64/128	32/64	16/16
P2TD	16/32	16/16	256/512	128/512	16/16
P3TD	4/4	4/8	8/8	8/8	4/4
P4CD	>1024	>1024	>1024	128/256	32/32
P4TD	>1024	>1024	>1024	>1024	64/128
P5CD	64/128	64/64	>1024	>1024	256/512
P5TD	256/256	128/128	>1024	>1024	>1024

**Table 4:** HC50/MIC ratio of polycationic polymers.

Polymer	*Enterococcus faecium*	*Staphylococcus aureus*	*Klebsiella pneumoniae*	*Pseudomonas aeruginosa*	*Enterobacter* spp.
P1TD	>256	>256	>128	>128	>256
P2CD	>64	>64	>16	>32	>64
P2TD	1	1	<1	<1	1
P3TD	<1	<1	<1	<1	<1
P4CD	<1	<1	<1	<1	<1
P4TD	<1	<1	<1	<1	16
P5CD	>16	>16	-^a^	-^a^	-^a^
P5TD	>4	>4	- ^a^	- ^a^	>4

^a^ HC50 and MIC values out of studied range. HC50/MIC could not be calculated.

## Data Availability

Not applicable.

## Supplementary Materials

The following supporting information can be downloaded at: https://www.mdpi.com/article/10.3390/pharmaceutics14112518/s1: Figure S1: Two-dimensional homonuclear correlation spectrum (COSY) of polymer P2C recorded in deuterochloroform. Figure S2: Two-dimensional heteronuclear correlation spectrum (HSQC) of polymer P2C recorded in deuterochloroform. Figure S3: Two-dimensional homonuclear correlation spectrum (COSY) of polymer P2T recorded in deuterochloroform. Figure S4: Two-dimensional heteronuclear correlation spectrum (HSQC) of polymer P2T recorded in deuterochloroform.
