# Supplementary material for: Tackling Antibiotic Resistance: Influence of Aliphatic Branches on Broad-Spectrum Antibacterial Polytriazoles against ESKAPE Group Pathogens
Source: Pharmaceutics. 2022 Nov 19;14(11):2518. doi: 10.3390/pharmaceutics14112518 (PMC9692804; doi:10.3390/pharmaceutics14112518)
Supplement: Supplementary file 1 [file pharmaceutics-14-02518-s001.zip › pharmaceutics-1970793-supplementary.pdf]

pharmaceutics

MDPI

Article

Tackling Antibiotic Resistance: Influence of Aliphatic Branches on Broad-Spectrum Antibacterial Polytriazoles against ESKAPE Group Pathogens

Cristian Rangel-Núñez [1], Inmaculada Molina-Pinilla [1], Cristina Ramírez-Trujillo [1], Adrián Suárez-Cruz [1], Samuel Bernal Martínez [2] and Manuel Bueno-Martínez [1,*]

[1] Departamento de Química Orgánica y Farmacéutica, Facultad de Farmacia, Universidad de Sevilla, C/Profesor García González 2, 41012 Sevilla, Spain
[2] Servicio de Microbiología, Hospital Universitario Virgen de Valme, 41014 Sevilla, Spain;
* Correspondence: mbueno@us.es

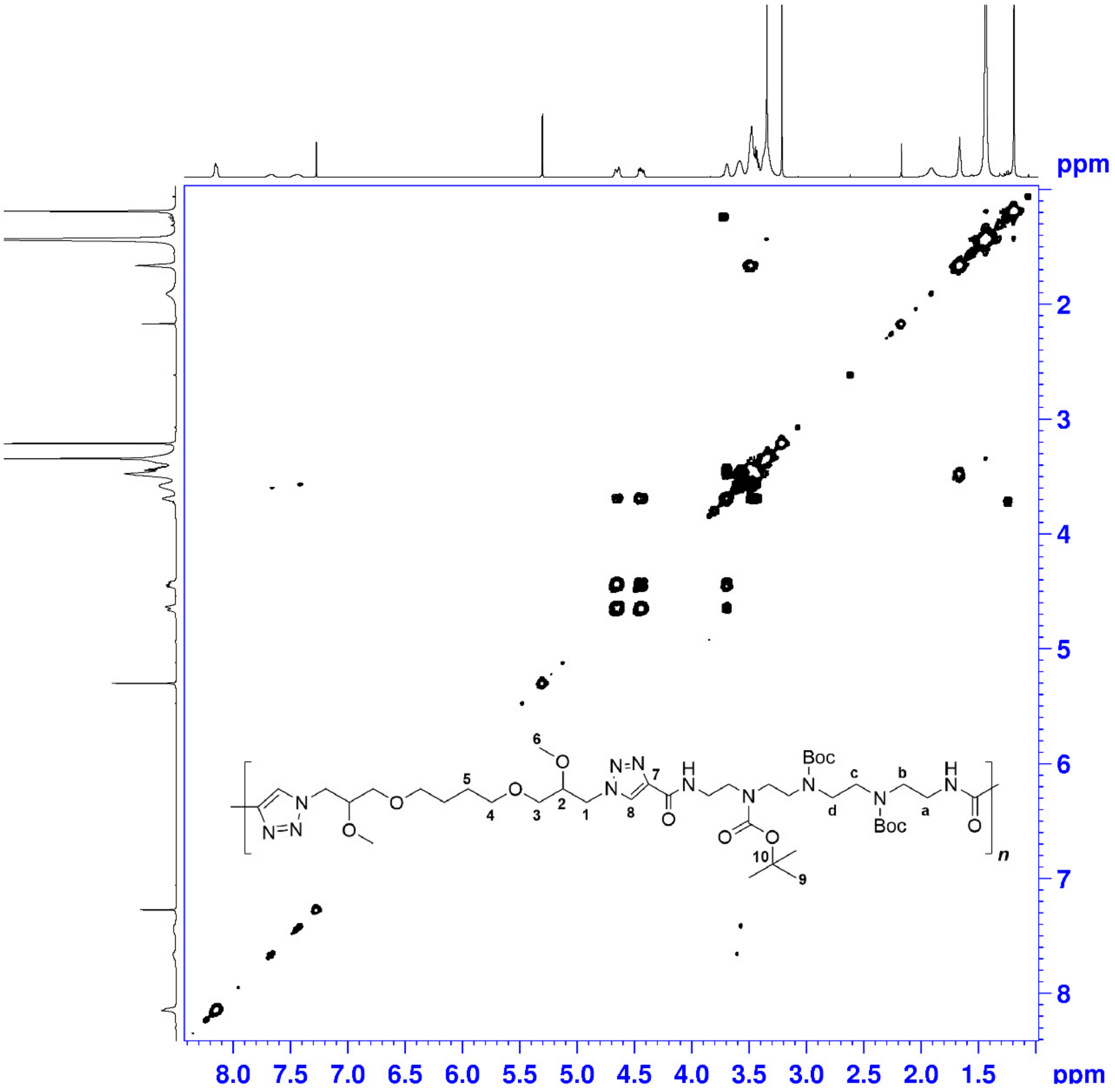

Figure S1. Two-dimensional homonuclear correlation spectrum (COSY) of polymer **P2C** recorded in deuterochloroform.

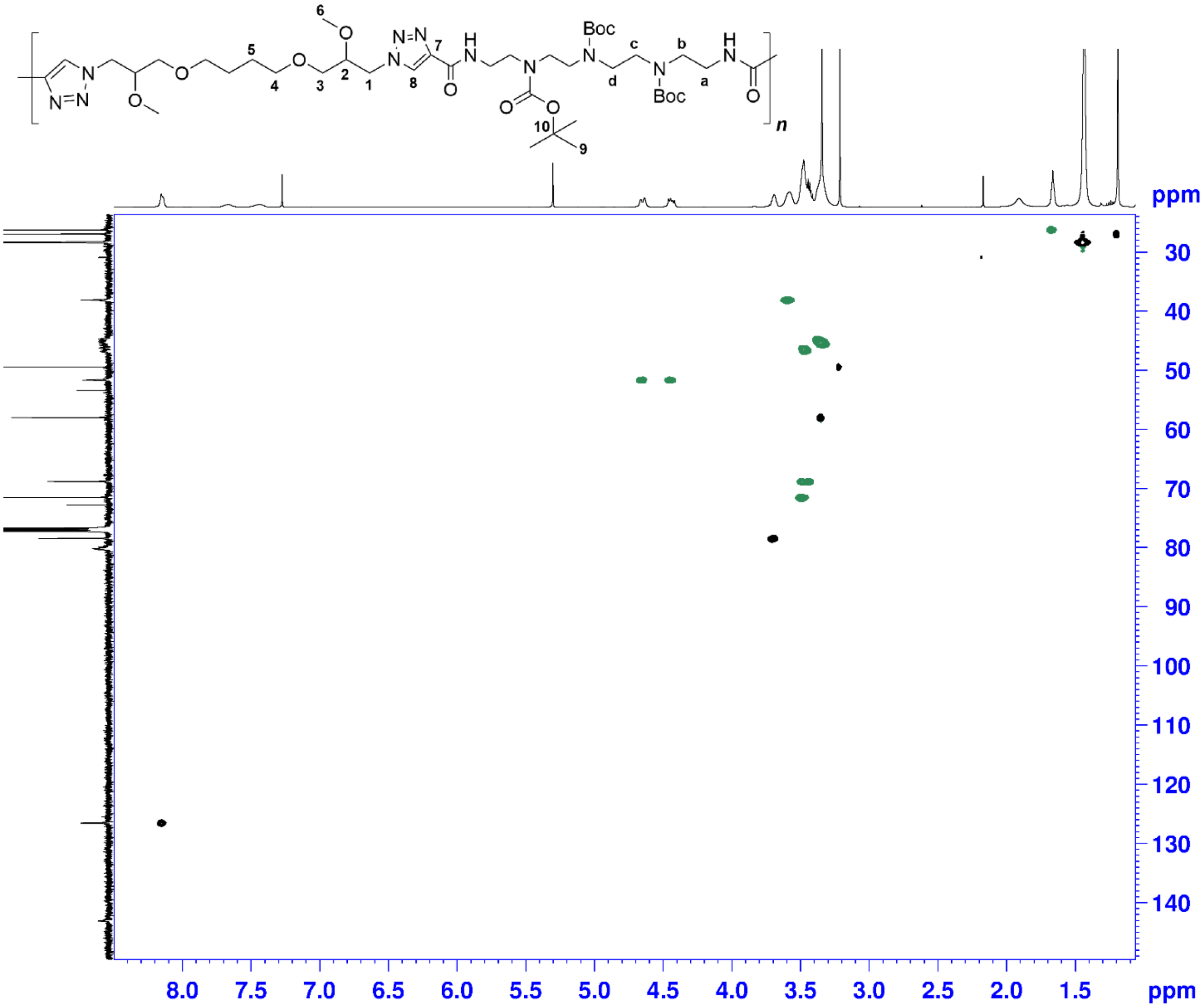

Figure S2. Two-dimensional heteronuclear correlation spectrum (HSQC) of polymer **P2C** recorded in deuterochloroform.

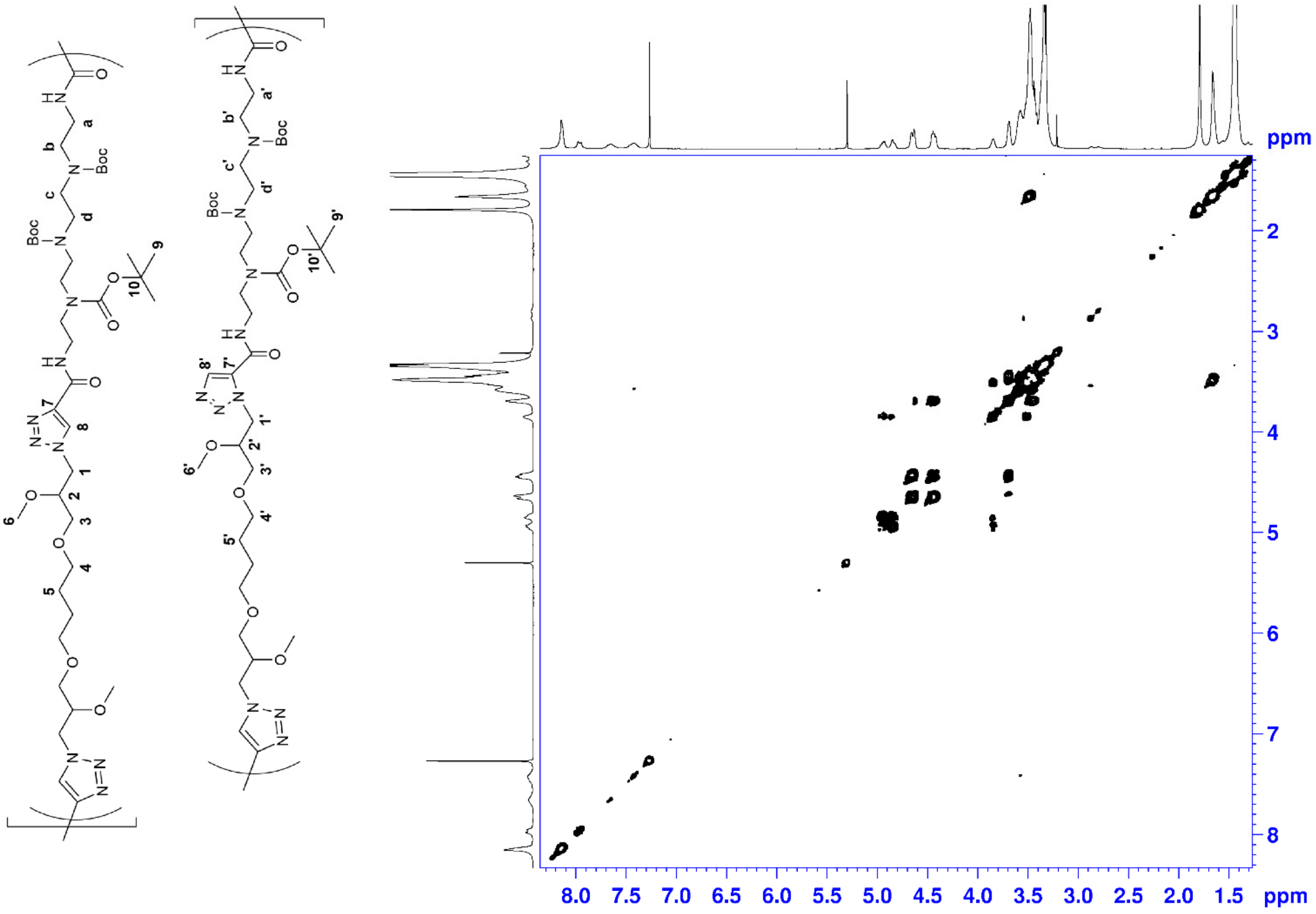

Figure S3. Two-dimensional homonuclear correlation spectrum (COSY) of polymer **P2T** recorded in deuterochloroform.

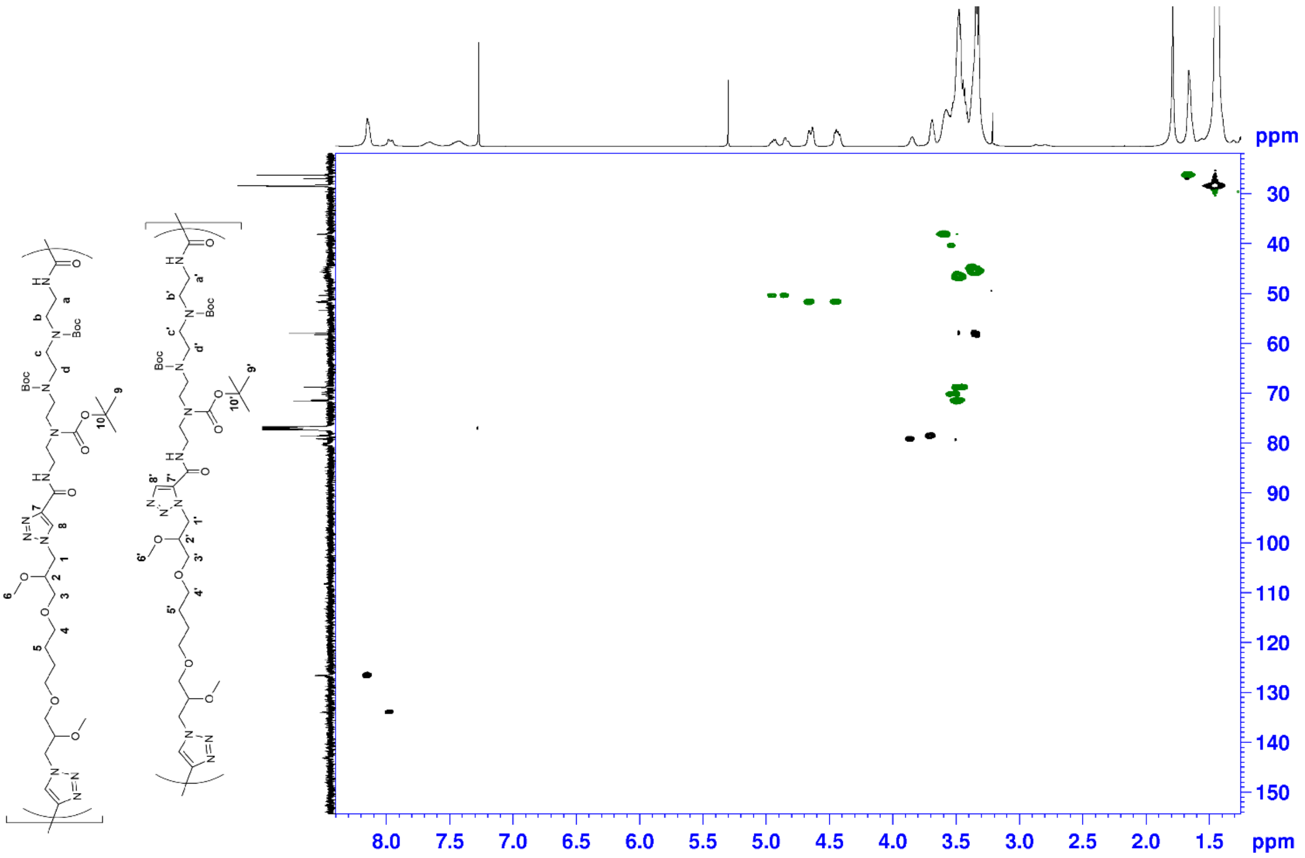

Figure S4. Two-dimensional heteronuclear correlation spectrum (HSQC) of polymer **P2T** recorded in deuterochloroform.
